# An HPLC method with diode array detector for the simultaneous quantification of chloroquine and desethylchloroquine in plasma and whole blood samples from *Plasmodium vivax* patients in Vietnam, using quinine as an internal standard

**DOI:** 10.1002/bmc.3657

**Published:** 2016-01-05

**Authors:** Toi Van Pham, Phuong Pham Nguyen, Tho Nguyen Duc Khanh, Nhien Nguyen Thanh Thuy, Ca Nguyen Thuy Nha, Thomas Pouplin, Jeremy Farrar, Guy E. Thwaites, Hien Tran Tinh

**Affiliations:** ^1^ Wellcome Trust Major Overseas Programme, Ho Chi Minh City‐In Partnership with Hospital for Tropical DiseasesOxford University Clinical Research UnitHo Chi Minh CityVietnam; ^2^ Centre for Tropical Medicine, Nuffield Department of Clinical MedicineUniversity of OxfordUK; ^3^ Mahidol‐Oxford Tropical Medicine Research Unit, Faculty of Tropical MedicineMahidol UniversityBangkokThailand

**Keywords:** HPLC‐DAD, SPE, chloroquine, desethylchloroquine, *Plasmodium vivax*, malaria

## Abstract

A sensitive, simple method for quantification of chloroquine (CQ) and desethylchloroquine (MCQ) in whole blood and plasma from *Plasmodium vivax* patients has been developed using HPLC with diode array detection (DAD). Solid‐phase extraction on Isolute‐96‐CBA was employed to process 100 μL of plasma/whole blood samples. CQ, MCQ and quinine were separated using a mobile phase of phosphate buffer 25 mm, pH 2.60–acetonitrile (88:12, *v*/*v*) with 2 mm sodium perchlorate on a Zorbax SB‐CN 150 × 4.6 mm, 5 μm column at a flow rate of 1.2 mL/min, at ambient temperature in 10 min, with the DAD wavelength of 343 nm. The method was linear over the range of 10–5000 ng/mL for both CQ and MCQ in plasma and whole blood. The limit of detection was 4 ng/mL and limit of quantification was 10 ng/mL in both plasma and blood for CQ and MCQ. The intra‐, inter‐ and total assay precision were <10% for CQ and MCQ in plasma and whole blood. In plasma, the accuracies varied between 101 and 103%, whereas in whole blood, the accuracies ranged from 97.0 to 102% for CQ and MCQ. The method is an ideal technique with simple facilities and instruments, bringing about good separation in comparison with previous methods. © 2016 The Authors Biomedical Chromatography Published by John Wiley & Sons Ltd

**Abbreviations used**
CQ: chloroquine
DAD: diode array detection
MCQ: desethylchloroquine
QN: quinine
SPE: solid‐phase extraction

## Introduction

*Plasmodium vivax* is the second most prevalent malaria in Vietnam and globally (Guerra *et al.*, 2010), representing 30% of all malaria infections (Doan *et al.*, 2006). For years, chloroquine (CQ) has remained the first‐line treatment for *P. vivax* malaria in most endemic countries other than Indonesia, Papua New Guinea, the Solomon Islands and Vanuatu, where artemisinin‐based combination therapies have been used since 2009 (Price *et al.*, 2011).

Chloroquine treatment failure in *P. vivax* by day 28 and prophylactic failure have been observed in Asia, Africa and Latin America (Baird, 2004). Although some studies indicated that CQ was an effective treatment for *P. vivax* in Vietnam (Taylor *et al.*, 2000), another study from 1997 to 2000 in Binh Thuan province, southern Vietnam, showed that 18 (16%) cases had late parasitological failure by day 28 (Phan *et al.*, 2002). Furthermore, the prevalence of CQ resistant *P. vivax* in other provinces of Vietnam such as Ninh Thuan was of concern (Huynh and Trieu, 2010).

CQ and its primary metabolite, mono‐desethylchloroquine (MCQ), are active against *P. vivax.* The quantification of their absorption in biological fluids is an important aspect to confirm the therapeutic efficacy. Resistance is defined when parasites are present in levels higher than the minimum effective concentration of the drug that has been routinely successful for elimination of the malaria parasite (Baird, 2009; WHO, 2009). According to WHO, the minimum effective concentration of CQ required in plasma to clear *P. vivax* parasites is 15 ng/mL. Moreover, the concentration of 70–90 ng/mL (CQ and MCQ) in whole‐blood is thought to result in suppression of chloroquine‐sensitive *P. vivax* (WHO, 2009). Therefore, determining concentrations of CQ and its metabolite (mono‐desethylchloroquine) in patients with *vivax* malaria plays an essential role in confirming the status of true CQ‐resistant *P. vivax*.

Several high‐performance liquid chromatography (HPLC) methods for quantification of CQ and its metabolites in whole blood, red blood cells, plasma and urine have been described (Akintonwa *et al.*, 1983; Baird *et al.*, 1997; Cheomung and Na‐Bangchang, 2011; Dua *et al.*, 1999; Karunajeewa *et al.*, 2008, 2010; Minzi *et al.*, 2003; Tang and Sojinu, 2012; Zuluaga‐Idárraga *et al.*, 2014). Most use liquid–liquid extraction with organic solvent, while some use solid‐phase extraction (SPE) for sample pre‐treatment (Chaulet *et al.*, 1993, 1994; Deng *et al.*, 2006; Lindegårdh *et al.*, 2002). The sample volume required for CQ and MCQ analysis varies from 500 to 1000 μL, using UV detection (Akintonwa *et al.*, 1983; Bergqvist and Frisk‐Holmberg, 1980; Karunajeewa *et al.*, 2008, 2010), diode array detection (DAD; Zuluaga‐Idárraga *et al.*, 2014) or fluorescence detection (Bergqvist and Frisk‐Holmberg, 1980; Dua *et al.*, 1999). Only 100 μL is required for the liquid chromatography tandem mass spectrometry system (LC‐MS/MS) (Boonprasert *et al.*, 2012; Tang and Sojinu, 2012). A method to facilitate the processing of samples from the field was developed whereby whole blood is dried onto the sampling paper for later quantification of anti‐malarial drugs (Blessborn *et al.*, 2006; Cheomung and Na‐Bangchang, 2011; Lindegårdh *et al.*, 2002).

A randomized controlled trial to assess and monitor the *in vivo* efficacy of dihydroartemisinin/piperaquine vs. CQ for the treatment of *P. vivax* infection and the *in vitro* susceptibility of *P. vivax* was conducted in Binh Phuoc province, Vietnam. One aim of this trial was to deepen understanding of *P. vivax* resistance in Vietnam. Pharmacokinetic investigations were planned to confirm and characterize true CQ resistance, which might play a critical role in shaping the treatment policy for the control of malaria.

In this article, we describe a sensitive and simple HPLC‐DAD method for quantification of CQ and MCQ in a small volume (100 μL) of human whole blood and plasma. The developed and validated method was subsequently applied to measure CQ and MCQ levels in samples collected from *P. vivax* patients of the above clinical trial. The method is optimal and relatively economical and requires only simple facilities and instruments. Time for analysis is short and only a small volume of sample is needed. Moreover, the initial results from fourteen patients with *P. vivax* malaria in Vietnam are a useful contribution to ongoing research on *vivax* malaria.

## Experimental

### Chemicals and reagents

All reagents and solvents used were of analytical grade. Potassium dihydrogen phophate (KH_2_PO_4_), phosphoric acid (H_3_PO_4_), formic acid (HCOOH), sodium perchlorate (NaClO_4_), sodium hydroxide (NaOH), acetonitrile (ACN) and methanol (MeOH) were purchased from Merck. Water was provided by a Purelab UHQ system (ELGA, Marlow, UK). The reference standards: CQ (98%, lot 047 K0038‐Sigma) and internal standard (IS) quinine (QN; 99.8%, lot 067 K1489‐Sigma), were purchased from Sigma‐Aldrich Singapore. Desethylchloroquine (MCQl lot BL 11088) was provided by the Walter Reed Army Institute of Research (Washington, DC, USA). Blank plasma samples were obtained from a pool of human plasma derived from people living outside the malaria endemic area (so chloroquine intake was most unlikely), supplied by the Blood Transfusion and Haematology Hospital in Ho Chi Minh City. Blank whole blood samples were provided by different donors who were healthy volunteers and gave consent forms for blood donation. They worked for Oxford University Clinical Research Unit (OUCRU) and did not take any tonic water that might contain quinine.

### Equipment

The liquid chromatography system was a Lachrom Elite–Hitachi (Merck) composed of an organizer, an autosampler L‐2200, two pumps L‐2130, a column oven L‐2350 and a diode array detector (DAD) L‐2455. The system was controlled by EZchrom Elite version 3.18 HPLC System Manager Software (Merck–Hitachi Japan). The analysis was performed on a Zorbax SB‐CN 150 × 4.6 mm, 5 μm equipped with 5 μm guard cartridges Zorbax SB‐CN 12.5 × 4.6 mm (Agilent Technologies, USA). The SPE was performed on Isolute‐96‐CBA 100 mg/2 mL 96 fixed well plates (Biotage AB, Uppsala, Sweden).

### HPLC analytical conditions

The mobile phase consisted of potassium dihydrogen phosphate buffer 25 mm, pH 2.60–ACN–sodium perchlorate 1 m (878:120:2, *v*/*v*/v), filtered (0.45 μm, RC membrane filter‐Sartorius) and degassed for 30 min. The chromatography was performed at ambient temperature (25 °C) for 10 min at a flow rate of 1.2 mL/min. The injection volume was 50 μL and the wavelength of DAD was set at 343 nm for CQ, MCQ and QN.

A system suitability test was performed prior to any sequence by injecting six consecutive aqueous standard solutions. The tolerated variation was assessed on area response and retention time with accepted variation of <2%.

### Standard solutions, calibration curves and QC samples

Stock solutions of CQ, MCQ and QN (1 mg/mL) were prepared in water. CQ and MCQ stock solution was further diluted with water to obtain fresh working solutions ranging from 0.2 to 100 μg/mL.

Plasma and whole blood calibration curve (CCs) and quality controls (QC) were prepared by diluting (1:20) the respective working solutions with blank plasma or whole blood to give nine CC points at 0, 10, 50, 100, 200, 500, 1000, 2000 and 5000 ng/mL (CQ, MCQ) and quality controls at low (QCL), medium (QCM) and high (QCH) concentrations of 40, 400 and 4000 ng/mL (CQ, MCQ).

### Sample preparation

For plasma samples, 1000 μL of IS [QN 200 ng/mL in phosphate buffer (PB) 25 mm pH 7.0] was added into 100 μL of thawed sample. The mixture was gently vortex‐mixed, rested for 2 min then centrifuged at 8000 rpm for 5 min.

For blood samples, 500 μL of ACN was added to the mixture of blood sample (100 μL) and pure water (100 μL), then carefully vortex‐mixed for 20 s, rested for 2 min before being centrifuged at 10,000 rpm for 5 min. The supernatant was aspirated into a new tube where 1000 μL of IS was added, then mixed and centrifuged at 8000 rpm for 5 min.

Following this, the SPE process was undertaken on the VAC‐Master 96 sample processing Manifold (IST‐Biotage, Sweden). The sample mixture was loaded on the Isolute‐CBA 96 fixed well plate (pre‐treated with 2 mL of ACN, 1 mL of PB 25 mm pH 7.0), then the plate was washed with 0.5 mL of PB 25 mm pH 7.0 and 1 mL of PB 25 mm pH 7.0–ACN (70:30, *v*/*v*). CQ and MCQ were eluted using 1.8 mL of ACN–formic acid (95:5, *v*/*v*) and collected in a 96 well collection plate; and then evaporated to dryness under stream air at 70 °C. Finally, the residue was reconstituted with 100 μL of the mobile phase prior to injection.

### Method validation

The full method validation of CQ and MCQ in human plasma and whole blood was performed in compliance with the US Food and Drug Administration (2001) bioanalytical method validation guidelines.

#### Specificity and selectivity

The ability of the method to differentiate the analytes (CQ, MCQ and IS/QN) towards endogenous plasma and whole blood interferences from at least six different donors was tested. A number of currently used medications like antimalarial drugs (dihydroartemisinin, piperaquine, mefloquine, artesunate, primaquine) and antipyretics (acetaminophen) which are likely to be administered to local patients were also tested.

#### Limit of detection and limit of quantification

The limit of detection (LOD) was defined as the concentration produced a signal three times higher than the noise of a blank sample. The analyte response at the limit of quantification (LOQ) should be at least five times the blank response, from the determination of six replicate spiked samples.

#### Linearity

The linearity of calibration consisted of nine calibration standards (including the blank sample) and was obtained by calculating the peak‐area ratios of CQ and MCQ to IS against the corresponding concentrations. The approach to choosing the best regression model was in accordance with the strategy proposed in the previous paper (Singtoroj *et al.*, 2006). Different regression models with or without data transformations and with different weightings were evaluated using model options in EZchrom Elite version 3.18 HPLC System Manager Software (Merck–Hitachi Japan). Back‐calculations were made to determine the concentrations of CQ and MCQ in the QC validation sets and clinical samples.

#### Recovery, accuracy and precision

The recovery yields for CQ, MCQ and IS (QN) were calculated at QCL, QCM and QCH by comparing the area response of spiked samples with that of unprocessed aqueous solutions. Intra‐day accuracy and precision were determined using five different replicates of QCL, QCM and QCH analyzed within the same day. Inter‐day accuracy and precision were estimated by analyzing five replicates of QC sets on four consecutive days. Intra‐ and inter‐day and total‐assay precisions were calculated using analysis of variance (ANOVA). Precision (%) was expressed as the mean relative standard deviation of the concentration area response (analytes/IS). Accuracy (%) was calculated as (estimated concentration/nominal value) × 100. The variation of the back‐calculated concentrations from the nominal concentrations should not be more than 15% in precision and range from 85 to 115% in accuracy (US Food and Drug Administration, 2001).

#### Matrix dilution effect

To investigate the matrix dilution effects, spiked samples (*n* = 6) were prepared at high concentration 8000 ng/mL (CQ and MCQ), over the upper limit of quantification (ULOQ), then diluted 10 times with blank matrix and processed as described in the sections on sample preparation and chromatography above.

#### Stability

The stability study was undertaken to test the stability of CQ and MCQ under different conditions, including heat evaporation at 70 °C, 24 h at room temperature (25 °C), 20 h in the autosampler (corresponding to the time between the first and the last sample analyzed by this method on a 96 well collection plate), three freeze–thaw cycles (−80 °C for at least 24 h per cycle) and long‐term stability (−20 °C for 1 month; −80 °C for 1, 3 and 6 months). All of the stability experiments were evaluated by comparing the CQ and MCQ concentrations area response (analytes/IS) of five different replicates of QCL and QCH against freshly prepared spiked solutions, and corrected from their initial values. Spiked solutions were considered stable if the deviation from the nominal values was within ±15% (US Food and Drug Administration, 2001).

### Application of the assay to clinical samples

The samples were collected from patients enrolled in a trial called ‘A randomized controlled trial to assess the antimalarial drug susceptibility and molecular characterization of *Plasmodium vivax* isolates in Vietnam’ (Institute of Malaria, Parasitology and Entemology of Ho Chi Minh City ethical approval 490/CV‐VSR, OXTREC reference 177–12 and the International Standard Randomised Controlled Trial number NCT01887821). The group of patients infected with *P. vivax* received CQ (25 mg base/kg for 3 days).

Blood was drawn in lithium heparin tubes on days 0, 7, 28 and any day of *P. vivax* recurrence. Whole blood and separated plasma (after centrifugation at 2000 ***g*** for 10 min) were collected and stored at the study site in liquid nitrogen, then transferred to the Pharmacology Lab‐OUCRU and kept at −80 °C until analysis. The clinical samples were analyzed and validated against freshly prepared CCs and QCs in either blank plasma or whole blood. The acceptance criterion on the validation QC sets was <15% for precision and between 85 and 115% for accuracy.

## Results and discussion

The serum threshold considered therapeutic against CQ‐susceptible parasites is set at 15 ng/mL (Baird *et al.*, 1997; WHO, 2009). As we expected to quantify low levels of CQ and MCQ, the method optimization focused on the LOD and LOQ using a small volume of samples (100 μL). In parallel, we managed to maximize the selectivity since we aimed to determine a single chromatographic condition to detect simultaneously CQ, MCQ and QN (IS) in plasma and whole blood.

### Sample preparation and optimization of chromatographic conditions

Because CQ, MCQ and QN are basic compounds with p*K*_a_ ≥ 10, ISOLUTE cation exchange sorbent–CBA was used to treat plasma and blood samples. Clean eluates with reasonable recovery ranging from >75% (whole blood) to >90% (plasma) for analytes (CQ, MCQ) and IS/QN were obtained in all QC levels.

**Figure 1:**
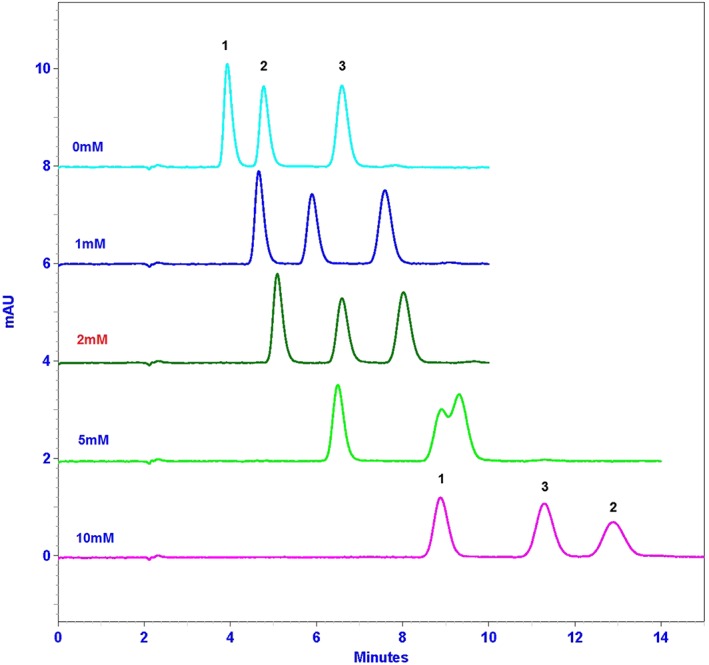
The chromatograms of analytes and IS as function of sodium perchlorate concentration in mobile phase (peak identification: 1, MCQ; 2, CQ; and 3, QN peak).

In preliminary experiments, separation was attempted on various reverse‐phase columns such as a Lichrospher RP_18_ end‐capped (125 × 4 mm, 5 μm) column, a Purospher RP‐8 end‐capped (125 × 4 mm, 5 μm) column and a Zorbax SB‐CN (150 × 4.6 mm, 5 μm) column; and various combinations of organic solvents (ACN) with phosphate buffers at different pH (ranging from 2.0 to 3.5). The best combination was the Zorbax SB‐CN 150 × 4.6 mm, 5 μm with KH_2_PO_4_ 25 mm–ACN (88:12, *v*/*v*). The pH of the buffer was also found to have a significant influence on the analytical performance in terms of specificity and sensitivity. At pH 2.0–2.3, the run time was shortened, but baseline separation was not achieved. We had to compromise and finally selected the buffer KH_2_PO_4_ 25 mm, pH 2.6–ACN (88:12, *v*/*v*) for the method validation. Additionally, sodium perchlorate 1 m was added to the mobile phase at different concentrations to achieve the best separation within reasonable time (10–14 min). The higher the concentration of sodium perchlorate in the mobile phase was, the better the separation of MCQ and CQ was. However, the separation between CQ and QN was not adequate and QN peak was shifted between MCQ and CQ peaks when sodium perchlorate concentration exceeded 10 mm. Therefore, the optimum sodium perchlorate concentration in the mobile phase was 2 mm, which allows a good separation of analytes within 10 min. Any further addition of sodium perchlorate resulted in worse separation between CQ and QN and extension of the run. The chromatograms with retention times of analytes and IS explaining the function of sodium perchlorate concentration in mobile phase are shown in the Fig. 1.

### Method validation

#### Specificity and selectivity

**Figure 2:**
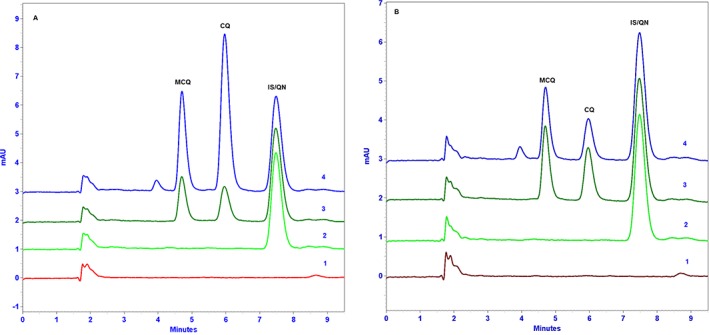
Chromatograms in whole blood (A) and plasma (B) at 343 nm. 1, Blank (without IS) sample; 2, blank sample with IS; 3, spiked samples with MCQ, CQ and IS; 4, clinical samples from patient 07MA‐016 at day 7.

The chromatograms CQ, MCQ and IS in human plasma and whole blood are shown in Fig. 2. The analytes were well defined and separated from matrix contaminants, with symmetrical shapes at the respective retention time of 4.62 min (CQ), 5.95 min (MCQ) and 7.50 min (QN/IS). Both analytes (CQ and MCQ) and IS were monitored at 343 nm but we also recorded the analysis from 200 to 400 nm to observe any suspected overlapping contaminants. In all batches of the validation, no interference from plasma or whole blood was found to co‐eluate with CQ, MCQ or the IS. Moreover, no interference from co‐medication (other antimalarial drugs and antipyretics) was noted at the retention time of analytes and IS. This was confirmed throughout the entire clinical sample analysis.

### Limit of detection and limit of quantification

The LOD and LOQ using 100 μL samples for CQ and MCQ were 4 and 10 ng/mL, respectively. There was no difference in the value of LOD and LOQ between plasma and whole blood samples.

In general, our results in terms of LOD, LOQ were comparable and more sensitive than those reported in a number of previous publications (Chaulet *et al.*, 1993, 1994; Cheomung and Na‐Bangchang, 2011; Dua *et al.*, 1999; Lejeune *et al.*, 2007; Yakasai, 2006; Zuluaga‐Idárraga *et al.*, 2014). Moreover, it was successfully achieved with a smaller sample volume of 100 μL vs. 150 μL (Cheomung and Na‐Bangchang, 2011), 200 μL (Bustos *et al.*, 2002), 500 μL (Dua *et al.*, 1999; Zuluaga‐Idárraga *et al.*, 2014) and 1000 μL (Chaulet *et al.*, 1994; Deng *et al.*, 2006; Karunajeewa *et al.*, 2008, 2010; Yakasai, 2006) in previous studies. However, when CQ and MCQ analysis was performed by LC‐MS/MS, the LOQ was 7.5 ng/mL for CQ using 200 μL plasma (Hodel *et al.*, 2009), 0.5 ng/mL for MCQ and 1.0 ng/mL for CQ (Tang and Sojinu, 2012), and 0.2 ng/mL for CQ and 0.4 ng/mL for MCQ using 100 μL plasma (Boonprasert *et al.*, 2012). Unfortunately, these methods require expensive instruments (LC‐MS/MS) and cannot be implemented in the ordinary analytical laboratories in developing countries, which are equipped with modest equipment. In such settings, the availability of facilities may be more important than method sensitivity.

#### Linearity

The calibration curve was linear over the range 10–5000 ng/mL for CQ and MCQ. The final regression model (log–log transformation with LSQ weighting 1/amount^2^ quadratic regression) resulted in small and evenly distributed residual errors and high coefficient of regression (*r* ^2^ > 0.999) for all of the CQ and MCQ calibration curves for both plasma and whole blood.

#### Recovery, accuracy and precision

The recoveries of CQ, MCQ and IS in plasma and whole blood were calculated at three QC levels (40, 400 and 4000 ng/mL) in five replicates, over 4 days. The mean recoveries of CQ and MCQ in all QC levels were 89.58–91.24 and 84.22–91.32% for plasma, and 77.74–82.06 and 75.88–79.76% for blood, respectively. Furthermore, the recovery of QN (IS) at the concentration of 200 ng/mL in all validation batches was 90.19% (RSD = 2.46%) for plasma and 91.05% (RSD = 4.17%) for whole blood.

The intra (*n* = 5) and inter‐assay (*n* = 20) coefficients of variation were <10% over all QC ranges for CQ and MCQ in plasma and whole blood samples. In plasma, the back‐calculated accuracies varied between 101 and 102% and between 102 and 103% at all QC levels for CQ and MCQ, respectively, whereas the accuracies ranged from 97.0 to 102% and from 98.5 to 102% in whole blood for CQ and MCQ respectively. The detailed results of the assay are presented in Table 1.

**Table 1:** Intra‐ and interday and total assay precision and accuracy for CQ, MCQ and QN (IS) in plasma and whole blood (ANOVA).

		Concentration (ng/mL)	Precision (CV, %)			Accuracy (%)	Recovery (%) (RSD)
			Inter‐assay (*n* = 5)	Intra‐assay (*n* = 20)	Total assay		
Plasma	CQ	40	2.50	2.18	2.24	102	89.58 (4.57)
		400	0.97	0.98	0.98	101	90.48 (1.74)
		4000	3.28	1.00	1.59	102	91.24 (1.92)
	MCQ	40	2.88	2.00	2.16	102	84.22 (3.26)
		400	1.39	0.66	0.82	102	90.04 (1.86)
		4000	4.36	1.13	2.02	103	91.32 (1.93)
Whole blood	CQ	40	3.79	3.49	3.54	101	82.06 (4.81)
		400	8.08	1.59	3.53	97.0	77.74 (7.14)
		4000	4.34	2.03	2.54	102	81.18 (8.01)
	MCQ	40	8.35	2.93	4.27	100	79.76 (5.07)
		400	9.83	1.87	4.27	98.5	75.88 (6.65)
		4000	4.61	1.44	2.26	102	76.60 (4.11)

CQ, Chloroquine; MCQ, desethylchloroquine; QN, quinine.

#### Matrix dilution effect

The results showed that relative standard deviations were <2% for MCQ and CQ in plasma and whole blood samples. The accuracies (± SD) were 98.9 ± 1.46 and 97.5 ± 1.61% in whole blood and 96.5 ± 0.90 and 99.4 ± 1.36% in plasma for MCQ and CQ, respectively. This indicated that samples containing high levels of CQ and MCQ over the upper limit concentration of the calibration curve (5000 ng/mL) could be diluted with blank matrix before the HPLC analysis.

#### Stability

Concerning the evaporation step under stream air at 70 °C, the mean recoveries of QCL and QCH were 97.5% (CV = 2.83%) and 96.7% (CV = 1.68%), and 100% (CV = 2.20%) and 94.7% (CV = 1.77%) for MCQ and CQ, respectively. For the stability of drugs in autosampler, the SPE eluates from spiked whole blood and plasma samples (*n* = 5) kept at ambient temperature in the autosampler were stable up to 20 h. This confirmed the possibility of analyzing a full 96 well plate within 20 h without any observed degradation from CQ, MCQ and the IS.

The results of the study also showed that plasma and whole blood samples containing CQ and MCQ at concentrations of 40 and 4000 ng/mL were stable after three consecutive freeze–thaw cycles, and when stored in a freezer at −20 and −80 °C for at least 6 months. The stability results are in agreement with other reported studies (Boonprasert *et al.*, 2012; Cheomung and Na‐Bangchang, 2011; Deng *et al.*, 2006; Dua *et al.*, 1999).

The variation in CQ and MCQ content was <10% from the initial values. The detailed results of stability of CQ and MCQ in plasma and whole blood are presented in Table 2.

**Table 2:** Variation of CQ and MCQ concentrations in plasma and whole blood under different storage conditions (*n* = 5).

Storage condition	Plasma				Whole blood			
	QCL		QCH		QCL		QCH	
	CQ (%)	MCQ (%)	CQ (%)	MCQ (%)	CQ (%)	MCQ (%)	CQ (%)	MCQ (%)
RT‐24 H	97.5	98.4	99.6	99.7	94.4	99.1	101	97.9
EXT‐20 H	101	101	99.2	99.5	95.1	102	100	101
C3	96.1	97.2	99.3	99.6	101	96.8	102	96.3
M1 (−20 °C)	100	100	100	103	97.5	96.7	99.4	90.4
M1 (−80 °C)	97.4	98.2	101	102	98.2	98.5	103	98.2
M3 (−80 °C)	97.5	97.7	98.4	99.5	97.9	99.5	99.0	95.1
M6 (−80 °C)	90.7	98.7	97.4	101	96.9	103	106	103

Results in percentage change = (mean value in stability sample/mean value in reference) × 100. Storage conditions are: RT‐24 H, unprocessed samples left at room temperature for 24 h;. EXT − 20 H, SPE eluates evaporated, reconstituted in mobile phase and kept at room temperature in autosampler for 20 h; C3, third freeze–thaw cycle; M1, storage for 1 month at −20 °C and −80 °C; M3, M6, storage at −80 °C for 3 and 6 months.

### Application of the assay to clinical samples from patients with *P. vivax*

The validated method was subsequently applied in the randomized controlled trial NCT01887821. The concentrations of CQ and MCQ were measured in plasma and whole blood from the *P. vivax* patients treated with CQ at a dose of 25 mg base/kg for 3 days.

**Figure 3:**
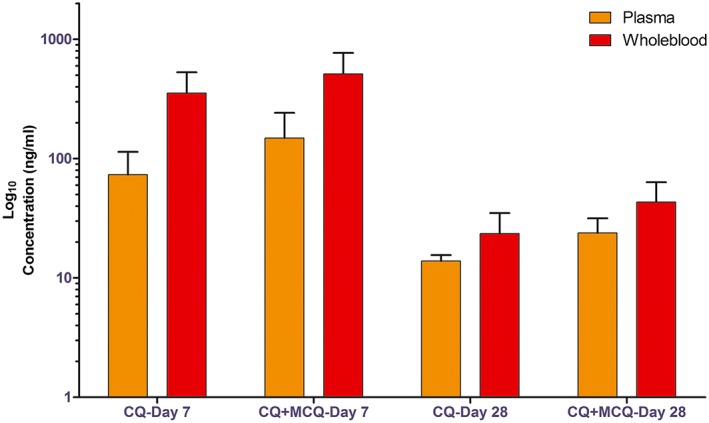
Concentration profile of CQ, CQ+MCQ in plasma and whole blood in 14 patients after the chloroquine treatment regimens on day 7, 28.

As mentioned above, CQ and MCQ are active against *P. vivax*, so the analyzed results present not only CQ and MCQ concentration separately but also the sum of (CQ + MCQ) concentration. The detailed results of CQ and combined (CQ + MCQ) measurements in blood and plasma samples collected on days 7 and 28 from 14 patients are presented in Fig. 3 and Table 3.

**Table 3:** Concentration (ng/mL) of CQ and its MCQ in plasma and whole blood samples from patients with *Plasmodium vivax*.

Patient ID.	Plasma						Whole blood					
	Day 7			Day 28			Day 7			Day 28		
	CQ	MCQ	[CQ + MCQ]	CQ	MCQ	[CQ + MCQ]	CQ	MCQ	[CQ + MCQ]	CQ	MCQ	[CQ + MCQ]
1	33.91	20.58	54.50	^a^	^a^	^a^	408.64	118.19	526.83	18.93	27.21	46.14
2	38.83	36.68	75.51	^b^	^a^	^a^	196.47	159.24	355.70	^a^	17.49	17.49
3	27.96	21.37	49.33	^a^	19.86	19.86	380.85	178.41	559.26	16.63	25.35	41.98
4	68.25	46.33	114.58	^a^	15.17	15.17	369.10	150.24	519.35	24.18	17.17	41.35
5	85.12	76.50	161.62	^a^	^a^	^a^	293.80	131.67	425.47	21.88	14.51	36.40
6	60.55	48.68	109.23	^a^	16.46	16.46	205.63	85.54	291.16	20.72	17.95	38.67
7	144.47	182.66	327.13	^a^	^a^	^a^	855.89	424.53	1280.41	53.18	45.08	98.26
8	118.55	127.82	246.37	^a^	25.25	25.25	507.78	191.14	698.92	12.84	17.45	30.28
9	75.78	80.97	156.75	^a^	24.53	24.53	456.79	185.91	642.70	34.13	29.95	64.08
10	44.98	69.13	114.11	^a^	^a^	^a^	214.22	109.29	323.51	14.97	19.47	34.44
11	156.72	179.72	336.43	15.07	22.89	37.96	320.66	143.12	463.78	16.11	11.59	27.70
12	79.85	90.79	170.63	12.72	15.02	27.74	333.48	151.84	485.32	36.61	23.88	60.48
13	44.12	37.19	81.30	^a^	^a^	^a^	211.86	90.85	302.72	14.91	12.49	27.40
14	48.71	35.55	84.26	^a^	^a^	^a^	198.56	105.03	303.59	20.72	20.77	41.48
*Mean*	*73.41*	*75.28*	*142.28*	*13.90*	*19.88*	*23.85*	*353.84*	*158.93*	*582.14*	*23.52*	*21.45*	*43.44*

a Below LOQ.

b Below LOD.

In general, large inter‐individual differences of CQ and MCQ levels were found in plasma as well as in whole blood, in agreement with previous publications (Dua *et al.*, 1999; Karunajeewa *et al.*, 2008, 2010; Sutanto *et al.*, 2010). All 14 subjects had CQ blood levels on day 7 of between 196 and 855 ng/mL, over the concentration of 100 ng/mL that is considered chloroquine‐resistant *P. vivax*. The CQ concentrations in whole blood were always higher than in plasma for all samples collected from these patients. The average ratios of CQ in whole blood/plasma in 14 patients were around 6.65 on day 7 and 3.35 on day 28, which is similar to the range noted in previous publication (Baird, 2004; Dua *et al.*, 1999).

The mean concentrations of CQ + MCQ combined at day 7 were 142 and 582 ng/mL, and at day 28 were 23.85 and 43.44 ng/mL for plasma and whole blood, respectively. These values indicate adequate drug exposure, since all are >15 ng/mL, the plasma threshold considered therapeutic against CQ‐susceptible parasites (WHO, 2009).

The total pharmacokinetic profile of CQ and MCQ and clinical results will be described in detail once the study is complete.

## Conclusions

This validated HPLC‐DAD method enables an accurate and selective quantification of CQ and its metabolite (MCQ) in plasma and whole blood samples. The method requires 100 μL of plasma or whole blood for LOD and LOQ of 4 and 10 ng/mL, respectively. Notably, the described method is simple and easy to apply for pharmacokinetic and drug monitoring studies where only modest facilities and instruments are available (HPLC‐UV or DAD). We hope that quantification of CQ and MCQ in plasma and whole blood from *P. vivax* patients in Vietnam will improve understanding of *P. vivax* resistance to CQ, which might contribute to shaping the treatment policy for the control of malaria in Vietnam and elsewhere.

### Authors' contributions

T.T.H., J.F., G.T., P.V.T., T.P. conceived and designed experiments. T.T.H., N.T.T.N., N.T.N.C., P.V.T., P.N.P., N.D.K.T. managed all participants and collected samples. P.V.T., P.N.P., N.D.K.T., T.P. performed the experiments and analysed the data. P.V.T., N.D.K.T., T.T.H., T.P. wrote the paper. All authors reviewed, approved and take responsibility for the content of the final manuscript.
